# Incidence and Age Distribution of Hospitalized Presumptive and Possible Abusive Head Trauma of Children Under 12 Months Old in Japan

**DOI:** 10.2188/jea.JE20180094

**Published:** 2020-02-05

**Authors:** Yui Yamaoka, Takeo Fujiwara, Yoshihisa Fujino, Shinya Matsuda, Kiyohide Fushimi

**Affiliations:** 1 Department of Health Services Research, University of Tsukuba, Ibaraki, Japan; 2 Center on Child Abuse and Neglect, University of Oklahoma Health Sciences Center, Oklahoma, USA; 3 Department of Global Health Promotion, Tokyo Medical and Dental University, Tokyo, Japan; 4 Department of Environmental Epidemiology, Institute of Industrial Ecological Sciences, University of Occupational and Environmental Health, Fukuoka, Japan; 5 Department of Preventive Medicine and Community Health, School of Medicine, University of Occupational and Environmental Health, Fukuoka, Japan; 6 Data Science Center of Occupational Health, University of Occupational and Environmental Health, Fukuoka, Japan; 7 Department of Health Policy and Informatics, Tokyo Medical and Dental University, Tokyo, Japan

**Keywords:** abusive head trauma, intracranial injury, ICD-10, incidence, Japan

## Abstract

**Background:**

Abusive head trauma (AHT) is the leading cause of fatal maltreatment among young children. The incidence of AHT in Japan, however, remains unknown. This study examined the incidence and distribution of age in months among young children under 12 months of age hospitalized with intracranial injury in Japan.

**Methods:**

We conducted a multicenter cross-sectional study of children under 36 months old admitted with intracranial injury to hospitals that employed the Diagnostic Procedure Combination (DPC) payment system between 2010 and 2013. Presumptive and possible AHT were defined using the combination of ICD-10 codes modified from the coding system recommended by the United States Centers for Disease Control and Prevention.

**Results:**

The average incidence was 7.2 (95% confidence interval [CI], 7.18–7.26) for presumptive and 41.7 (95% CI, 41.7–41.8) for possible AHT per 100,000 children less than 12 months old from 2010 to 2013. The distributions of age in months for both presumptive AHT and possible AHT had peaks at around 2 and 8 months.

**Conclusions:**

This is the first study to report the incidence of hospitalized children with presumptive and possible AHT using population-based data. Further datasets are needed to evaluate the incidence and specific preventive strategies to prevent AHT in infants during the months of highest risk.

## INTRODUCTION

Abusive head trauma (AHT), or shaken baby syndrome (SBS), is the leading cause of death due to child maltreatment^1^^,^^2^ and may lead to neurological sequelae.^3^ To evaluate prevention strategy, the incidence of AHT needs to be reported repeatedly. Using active surveillance, the incidence of admitted AHT children (per 100,000 children) was reported from 12.5 to 38.8 in several Western countries, such as United States (children under 1 year old),^4^^,^^5^ United Kingdom (children under 2 years old),^6^ Australia (children under 2 years old),^7^ or New Zealand (children under 2 years old).^8^ These active surveillance systems are expensive, however, and recent studies have demonstrated the validity of passive surveillance systems using the algorithm of International Classification of Diseases, Tenth Revision (ICD-10) codes for hospitalization records.^9^^,^^10^

Although the epidemiology of AHT shares similarity among countries, it has not been sufficiently examined in Japan. The only “self-reported” prevalence for shaking was reported as 3.9% in a population-based study,^11^ which was similar to that in Western countries. However, there are few studies that examine the incidence of AHT using hospitalization records in Japan.

Japan has a unique health care system and medical facilities. Universal health coverage was established in 1958,^12^ and everyone is insured and has unlimited access to all health care facilities at a relatively low cost. In addition, Japan has one of the highest penetration rate of computed tomography (CT) and magnetic resonance imaging (MRI) in developed countries.^13^ The number of CT and MRI scans was approximately three- to four-fold higher than the average for Organization for Economic Co-operation and Development (OECD) countries. Thus, Japanese hospitalization records may have the potential capacity to detect intracranial injury among young children. A nationwide study investigating the incidence of admitted AHT is necessary to confirm the similarity of the incidence of AHT regardless of health care system or medical equipment.

Furthermore, Fujiwara et al^14^ reported that the prevalence of AHT diagnosed with CT showed two peaks: around 2 to 4 months and around 7 to 9 months. The second peak around 7 to 9 months is not clearly reported in other countries (ie, United States,^15^^–^^17^ Scotland,^18^ or China^19^), and the mechanisms and preventive strategies for later infancy remain unknown. Since study subjects in the previous study are limited to a single national central hospital,^14^ the distribution of ages in month for AHT using nationwide samples with hospital-based data need to be confirmed. Therefore, this study examined the incidence of hospitalized AHT for infants under 12 months old and described the distribution of ages in month for AHT children under 36 months old using hospital-based medical claim data.

## METHODS

### Data source

This study utilized the Diagnosis Procedure Combination (DPC) database, which includes patient discharge and administrative claims.^20^ The DPC database does not include cases due to motor vehicle accidents because administrative claims do not cover such. All advanced treatment hospitals have been directed to adopt the DPC in 2003, and other general hospitals have adopted it voluntarily. Advanced treatment hospitals have capabilities to provide advanced medical care and technologies with 400+ beds and 16+ clinical departments. The number of participating hospitals increased from 82 hospitals with 68,982 inpatient beds in 2003 to 1,585 hospitals with 492,206 inpatient beds in 2014. This number includes more than half of all general hospital beds nationwide.^21^ The DPC database includes the demographics of patients, diagnosis, and comorbidities using the ICD-10 codes, treatments and surgeries, length of stay, and discharge status.^22^^,^^23^ Physicians in charge must code the diagnosis according to medical records, and medical clerks or licensed medical information managers record all drugs, devices, and procedures during the admission.^24^

### Identifying suspected AHT

This study targeted children under 36 months old who were admitted to a hospital in the DPC database due to intracranial injury from 2010 through 2013. We extracted children with suspected AHT who had at least one diagnosis from multiple categories of disease diagnosis (“main diagnosis,” “admission-precipitating diagnosis,” “most resource-consuming diagnosis,” “second most resource-consuming diagnosis,” “comorbidities present at time of admission,” and “conditions arising after admission”).^22^^,^^23^ We defined AHT based on certainty of AHT, that is, “presumptive AHT” definitions following the recommendation by the United States Centers for Disease Control and Prevention (CDC)^9^^,^^25^ and “possible AHT” definition instead of “probable AHT” used in previous studies^5^^,^^9^^,^^10^^,^^27^ as shown in Table 1. We decided to use the term “possible” AHT in this study because DPC data did not clearly have codes for unintentional injuries. The considerations for these differences among definitions are mentioned in the discussion section. Presumptive AHT represented children who had intracranial injuries (ICD-10 intracranial injury code: S06.0–S06.9, T90.5) and either an intentional injury codes (physical abuse: T74.1, other maltreatment syndromes, T74.8, maltreatment syndrome, unspecified, T74.9, assault: Y00, Y01, Y04, Y08, Y09; other maltreatment; Y07.0, Y07.1, Y07.2, Y07.3, Y07.8, Y07.9) or retinal hemorrhage (H356), without any kind of exclusion criteria for plausible causes including coagulation defects, purpura and other hemorrhagic conditions (D65–D69), deficiency of vitamin K (E56.1), birth trauma (P10–P15), intracranial nontraumatic hemorrhage of the fetus and newborn (P52), and hemorrhagic disease of the fetus and newborn (P53). Possible AHT included, in addition to presumptive cases, children who had intracranial injury (S06.0–S06.9, T90.5) without unintentional injury code (transport accident [V01–V99], other external causes of accidental injury including falls [W00–X59], sequelae of transport accident [Y85], and sequelae of other accidents [Y86]), applying the above excluding criteria (eg, coagulation defects). If the children were admitted multiple times in the same year, we selected one admission due to presumptive AHT; poorer outcome at discharge, if multiple diagnoses were the same (presumptive or possible); or a younger age, if the other conditions were the same.

**Table 1.** International Classification of Diseases, 10^th^ version (ICD-10) codes to identify presumptive and possible abusive head trauma (AHT)

ICD-10 Code		Description
1. Intracranial injury codes	S06.0–S06.9	Intracranial injury Sequelae of intracranial injury classifiable to S06
	T90.5	

2. Intentional injury codes	T74.1, T74.8, T74.9	Physical abuse; Other maltreatment syndromes; Maltreatment syndrome, unspecified
	Y00, Y01, Y04, Y08, Y09	Assault
	Y07.0, Y07.1, Y07.2, Y07.3, Y07.8, Y07.9	Other maltreatment

3. Retinal hemorrhage	H35.6	Retinal hemorrhage

4. Unintentional injury codes	V01–V99	Transport accident
	W00–X59	Other externa cause of accidental injury (including falls)
	Y85–Y86	Sequelae of transport accident; Sequelae of other accidents

5. Excluding codes	D65–69	Coagulation defects, purpura and other hemorrhagic conditions
	E56.1	Deficiency of vitamin K
	P53	Hemorrhagic disease of the fetus and newborn
	P10–P15	Birth trauma
	P52.8	Other intracranial (nontraumatic) hemorrhages of the fetus and newborn
	P52.9	Intracranial (nontraumatic) hemorrhage of the fetus and newborn, unspecified

Presumptive AHT cases were [Intracranial injury codes] AND [Intentional injury codes AND/OR Retinal hemorrhage] WITHOUT [Excluding codes].

Possible AHT cases were presumptive AHT cases plus [Intracranial injury code] WITHOUT [Unintentional injury codes AND/OR Excluding codes].

### Analysis

We described each annual number and the proportion of total admissions, intracranial injury admissions, possible and presumptive AHT at the age of 0–11, 12–23, and 24–35 months old. The number of hospitals that have adopted the DPC system increased year by year, and the number of infants less than 12 months old admitted to DPC hospitals increased from 147,776 in 2010 to 176,956, 206,681, and 203,301 in 2011, 2012, and 2013, respectively. Therefore, the number of infants as the denominator was adjusted by the number of births each year multiplied by the ratio of the increase of admissions to DPC hospitals in each year compared with that of in 2013. In addition, we took into account the coverage rate of the DPC system among hospitals nationwide. The formula for calculating incidence in each year for children under 12 months old is:

$$\begin{array}{l} \text{Incidence of presumptive AHT }(\text{y})\\ \;=\frac{number\;of\;presumptive\;AHT\;(y)}{number\;of\;infant\;population\;in\;Japan\;(y)\times \frac{total\;number\;of\;infant\;cases\;in\;DPC\;(y)}{total\;number\;of\;infant\;cases\;in\;DPC\;in\;2013}}\times \frac{1}{Z} \end{array}$$

here, (y) represents each year, and Z represents the proportion of DPC introduced hospitals among hospitals having capabilities to provide advanced pediatric care, such as advanced treatment hospitals, children’s hospitals, and related institutions.

In addition to 83 advanced treatment hospitals, there were 36 children’s hospitals, which belonged to the Japanese Association of Children’s Hospitals and Related Institutions (JACHRI). These hospitals can provide comprehensive pediatric care with 100+ pediatric beds, 20+ pediatricians per 100 beds, and CT/MRI facilities. Twenty-five hospitals (69.4% of children’s hospitals and related institutions) and all 83 advanced treatment hospitals employed the DPC payment system. We assumed that all the AHT admission cases would be captured in these advanced treatment hospital and JACHRI. In total, 108 (ie, 83 plus 25) out of 119 (ie, 83 plus 36) advanced treatment hospitals and children hospitals and related institutions were covered in the DPC payment system (90.8%), which is the same during the observed period (ie 2010–2013). Therefore, we used the coverage rate (Z) as 90.8% for all years.

Next, we examined the differences between presumptive and possible AHT cases among children under 36 months old. We compared demographics (ie, sex, age), clinical conditions (ie, Japan Coma Scale [JCS], duration of admission, transfer from other hospital, use of ambulance), outcome at discharge with six categories (ie, cured, improved, remission, remain unchanged, exacerbation, and death), and admission cost. We dichotomized outcomes into poor (remain unchanged, exacerbation, or death) and better (cured, improved, or remission) outcomes. Under the DPC payment system, the total admission cost, which is based on diagnosis group, includes basic hospital stays and any treatments during the admission, such as laboratory tests, medications, injections, and surgical procedures. In addition, for sensitivity analysis, the month-age distribution of epidural hemorrhage (EDH; S06.4) was compared between presumptive AHT, possible AHT and possible AHT excluding EDH, because EDH is more likely to occur after falls.^26^ Lastly, we described the distribution of age in months for presumptive AHT, possible AHT, and possible AHT excluding EDH. We used STATA MP, version 14.0 (Stata Corp LP, College Station, TX, USA). The ethical review board at Tokyo Medical and Dental University approved this study.

## RESULTS

Table 2 shows the number of admissions with presumptive and possible AHT among children under 36 months old from 2010 through 2013. The number of total admissions of children increased from 2010 through 2013 because the number of hospitals enrolled in the DPC database rose year by year. Among presumptive AHT admissions, the majority were infants (61.4–81.6%), and the proportion of older children was relatively small. Among possible AHT, almost half of them were infants; however, the proportion of children aged 24–35 months old increased from 23.2% to 25.5%. Among possible AHT, 11.1% to 13.5% were presumptive AHT.

**Table 2.** Admissions with presumptive and possible abusive head trauma (AHT) among children under 36 months old in 2010–2013

	2010		2011		2012		2013	
	*n*	%	*n*	%	*n*	%	*n*	%
Total admission								
0–11 months old	147,776	59.2%	176,956	59.2%	206,681	59.3%	203,301	60.3%
12–23 months old	63,637	25.5%	77,592	26.0%	91,401	26.2%	85,925	25.5%
24–35 months old	38,160	15.3%	44,242	14.8%	50,680	14.5%	48,189	14.3%
Total	249,573		298,790		348,762		337,415	

Presumptive AHT								
0–11 months old	35	61.4%	59	79.7%	80	81.6%	72	75.8%
12–23 months old	7	12.3%	9	12.2%	13	13.3%	14	14.7%
24–35 months old	15	26.3%	6	8.1%	5	5.1%	9	9.5%
Total	57		74		98		95	

Possible AHT								
0–11 months old	273	54.9%	367	55.3%	401	55.3%	384	53.6%
12–23 months old	100	20.1%	143	21.5%	151	20.8%	150	20.9%
24–35 months old	124	24.9%	154	23.2%	173	23.9%	183	25.5%
Total	497		664		725		717	

**Figure 1.**
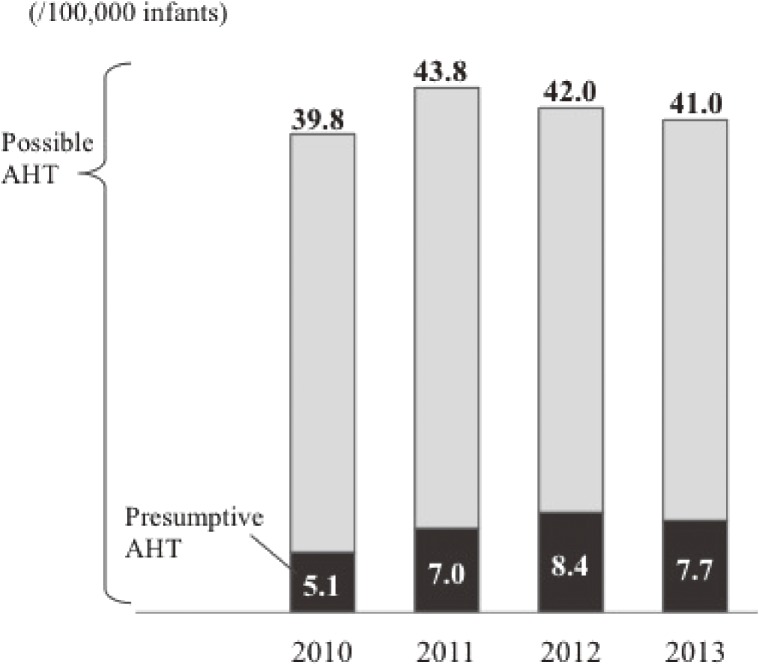
Incidence of presumptive and possible abusive head trauma (AHT) per 100,000 infants between 2010 and 2013

Table 3 and Figure 1 shows the incidence of presumptive and possible AHT per 100,000 infants, adjusted by the changes in the number of admissions in DPC hospitals from 2010 through 2013 and the coverage of DPC system among hospitals where can provide advanced pediatric treatment. The overall incidence was 7.22 (95% confidence interval [CI], 7.18–7.26) per 100,000 infants for presumptive AHT, 41.7 (95% CI, 41.7–41.8) per 100,000 infants for possible AHT. The incidence for presumptive AHT increased (*P* for trend = 0.042), but the incidence of possible AHT was stable between from 2010 through 2013 (*P* for trend = 0.950).

**Table 3.** Incidence of presumptive and possible abusive head trauma (AHT) in children under 12 months of age

Year	2010	2011	2012	2013	Total (2010–2013)
Number of Presumptive AHT (*a*)	35	59	80	72	246
Number of Possible AHT (*a*)	273	367	401	384	1,425
Infant population (*c*)	1,037,633	1,060,000	1,032,000	1,030,000	4,159,633
Number of admissions in DPC hospitals (*d*)	147,776	176,956	206,681	203,301	734,714
Ratio to the number of admissions in DPC hospitals compared to that of 2013 (*d*/*e*)	0.73	0.87	1.02	1.00	
Adjusted infant population (*b* = c*d/*e*)	754,238	922,639	1,049,158	1,030,000	3,756,035
**Incidence of presumptive AHT (/100,000 infants)** (*a*/b*1/*f*)	5.1	7.0	8.4	7.7	7.2
**Incidence of possible AHT (/100,000 infants)** (*a*/b*1/*f)*	39.8	43.8	42.0	41.0	41.7

DPC, *Diagnosis Procedure Combination.*

*a.* N of AHT (*year*): Number of presumptive AHT of infants in each year.

*b.* Adjusted N of infant (*year*): Number of infant population each year adjusted by the increase of DPC hospital admissions compared to 2013.

*c.* N of infant (*year*): Number of infant population in each year.

*d.* N of DPC (*year*): Number of total infant admissions in each year.

*e.* N of DPC (*2013*): Number of total infant admissions in 2013.

*f*. Coverage of DPC system among advanced treatment hospitals and children hospitals (90.8%).

Table 4 shows the differences between children less than 36 months age admitted with presumptive and possible AHT. Children with presumptive AHT were younger, had a poorer JCS score, longer hospitalization, were more often transferred from another hospital, more often used an ambulance, and showed poorer outcome at discharge than children with possible AHT. The proportion of EDH was higher among possible AHT cases than in presumptive AHT (18.8% vs 6.8%). The medians of the admission costs were JPY 754,000 (approximately USD 7,540) and JPY 240,000 (approximately USD 2,400) for presumptive and possible AHT per case, respectively.

**Table 4.** Characteristics of young children under 36 months old admitted with presumptive and possible abusive head trauma (AHT)

		Presumptive AHT (*n* = 324)		Possible AHT (*n* = 2,603)	
		*n*	%	*n*	%
Sex	Male	200	61.7%	1,553	59.7%
	Female	124	38.3%	1,050	40.3%
Age, years	0	246	75.9%	1,425	54.7%
	1	43	13.3%	544	20.9%
	2	35	10.8%	634	24.4%
Japan Coma Scale at admission	0	217	67.0%	1,783	68.5%
	1	57	17.6%	531	20.4%
	2	38	11.7%	245	9.4%
	3	12	3.7%	44	1.7%
Duration, days	Median, IQR	15	7–29	4	2–10
	(range)	(2–328)		(2–752)	
Transfer from other hospital	No	198	61.3%	1,811	69.7%
	Yes	125	38.7%	786	30.3%
Ambulance	No	191	59.1%	1,320	50.8%
	Yes	132	40.9%	1,277	49.2%
Poor outcome^a^	No	281	91.2%	2,447	96.0%
	Yes	27	8.8%	103	4.0%
Epidural hemorrhage	No	302	93.2%	2,114	81.2%
	Yes	22	6.8%	489	18.8%
Total admission cost (1,000 yen ≓ 10USD)	Median, IQR	754	373–1929	240	119–582
	Mean, SD	1,468	2007	699	1372
	(range)	(6.3–17,400)		(1.5–17,400)	

IQR, interquartile range.

^a^Poor outcome represented remain unchanged, exacerbation, and death.

**Figure 2.**
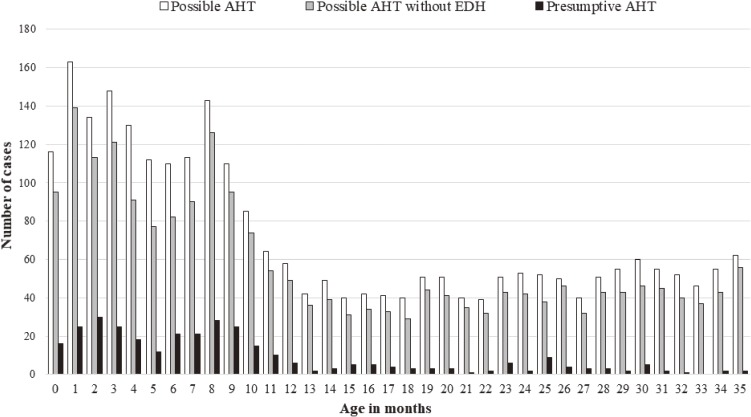
The distribution of age in months for presumptive abusive head trauma (AHT), possible AHT without epidural hemorrhage (EDH), and possible AHT

Figure 2 shows the distribution of age in months for presumptive AHT, possible AHT, and possible AHT excluding EDH. For both presumptive and possible AHT, we observed two peaks, at 1–3 and 7–9 months old. Further, possible AHT excluding EDH cases also had two peaks at 1–3 and 7–9 months old. After 12 months old, the number of both presumptive and possible AHT cases became relatively stable.

## DISCUSSION

This is the first study to describe the incidence of presumptive and possible AHT using ICD-10 codes for admissions in Japan. The incidences were 7.2 for presumptive AHT and 41.7 for possible AHT per 100,000 infants less than 12 months old from 2010 through 2013, Both presumptive and possible AHT had two peaks, at 1–3 and 7–9 months old.

### Incidence of presumptive AHT

The incidence of presumptive AHT was comparatively lower than that of previous studies, which was 13.0–33.4 per 100,000 infants based on ICD-9 or 10 codes in other counties.^4^^,^^5^^,^^8^^–^^10^^,^^18^^,^^27^ The definitions of AHT to estimate incidences varied among studies. The passive surveillance to capture AHT cases from claim data was obtained from the hospital discharge records used in several studies.^5^^,^^9^^,^^10^^,^^27^ Berger et al reported a high accuracy of the CDC operational definition of AHT using ICD-9CM codes, compared with AHT defined by child protection teams.^28^ However, Japanese coders may not have sufficient training in coding for cause of intentional injury, such as physical abuse (T74.1, T74.8, T74.9), assault (Y00, Y01, Y04, Y08, Y09), or other maltreatment syndromes (Y07.0, Y07.1, Y07.2, Y07.3, Y07.8, Y07.9) that were used in other studies.^5^^,^^9^^,^^10^^,^^27^ Therefore, the insufficient coding for intentional injury might lead to underestimate the incidence of presumptive AHT in Japan. On the other hand, the incidence of possible AHT in this study indicated the possibility of AHT cases at most, because we only could obtain AHT without unintentional injury code. It might include some intracranial injuries that physicians or coders did not code for the cause of injury. Therefore, to obtain accurate incidence for AHT and to establish effective passive surveillance, sufficient education for physicians and coders is needed to ensure the effective evaluation of AHT.

Another method to obtain better estimates for AHT is to utilize active surveillance with multiple sources of information.^4^^,^^8^^,^^18^ For example, Keenan et al^4^ abstracted information from medical charts and medical examiner records, reviewed radiological examinations, and utilized determination of AHT by social service agency. Because AHT is a rare event and a large population or multiple years of followup is needed to assess its incidence, conducting active surveillance becomes expensive and time-consuming. Previous studies using passive surveillance showed similar incidence of AHT with that of active surveillance.^9^^,^^10^ Therefore, improving better passive surveillance is epidemiologically essential to assess the incidence of AHT. From a practitioner’s point of view, detailed information from medical records and child protection services would be helpful for better understanding and prevention of AHT. There are both needs to establish passive and active surveillances that capture the occurrence of AHT in hospitals across the country and share information from multidisciplinary organizations.

### Characteristics of presumptive AHT

This study revealed that the children with presumptive AHT were younger, had severe medical conditions at hospitalization and at discharge, EDH was less frequent, and higher admission cost than possible AHT cases. Longer hospitalization,^3^ poor outcomes,^3^^,^^29^ and higher admission cost^30^ have also been reported as characteristics of AHT. In terms of clinical presentations, seizures at presentation or within 24 hours, and apnea at presentation were significant characteristics of AHT that could lead to a severe JCS score,^31^ as seen in presumptive AHT in our study. Therefore, we can confirm that our study subjects had similar clinical severities to that of presumptive AHT cases, and not possible AHT, in the other studies.

### The distribution of age in months

We found two peaks for both presumptive and possible AHT, at 1–3 and 7–9 months old. The second-peak distribution in 7–9 months was similar to that of possible AHT without EDH, suggesting that both presumptive and possible AHT during 7–9 months may not have been caused by direct impact, such as falls. A plausible explanation for the first peak at 2 months for presumptive and possible AHT is “the crying period”, which is same as in previous studies.^15^^,^^32^ The possible interpretations for the second peak at 8 months for presumptive AHT need further consideration. This second peak has not been reported in other countries, and is consistent with a previous study conducted by identifying AHT using CT in Japan.^14^ Other mechanisms need to be considered for presumptive AHT in late infancy. One possible explanation is that different types of parenting stress or challenges occur in the later months of infancy, for example, the transition from milk to solid food^33^; the increased supervision needed as a result of developmental milestones, such as crawling or pulling up^34^; or anxiety in regard to the delay of those development milestones. Even though Japan has easy access to radiographic examination, as it has the highest rates of CT and MRI in developed countries as well as universal health insurance coverage, the second peak at 8 months for AHT is unique and its underlying mechanisms remain unclear. Further research is needed to target presumptive AHT, especially in the later months of infancy, to determine the possible reasons and preventive strategies in Japan.

### Limitations and future direction

There are several limitations and challenges for further research. First, we utilized the DPC database, which covered more than half of all general hospital beds nationwide. All university hospitals and all 34 children’s hospitals and related institutions that provide specialized comprehensive pediatric care were included. We might, however, have missed AHT cases admitted to general hospitals, cases that died before transfer to pediatric tertiary hospitals, or cases that died at home. Moreover, we could not distinguish the children who were admitted or who had transferred hospitals over the years, although we can track the children who were admitted multiple times in the same year. Therefore, future research should utilize the national database for claim records and track patients over years and between hospitals. Second, the incidence of presumptive AHT in this study may have been underestimated due to poor training for physicians and coders to code intentional injury. A multidisciplinary approach for information integration may help to accurately capture the cause of injury in DPC data. Lastly, this study examined the incidence of AHT in population-based samples obtained from the DPC database. However, the DPC database did not include patients who died in the emergency department without being admitted to the hospital. There is a possibility that fatal presumptive AHT cases are underestimated. Information from forensic autopsy records must be added to include fatal AHT cases to determine a more accurate incidence in the future.

### Conclusion

This is the first study to report the incidence among hospitalized children with presumptive and possible AHT under 12 months old in Japan, which was 7.2 and 41.7 per 100,000 infants, respectively. Two peaks for distributions of age in months among children under 36 months old were found at 1–3 and 7–9 months old, a finding which is unique to the Japanese population. Further active surveillance is needed to validate the accuracy of passive surveillance using DPC data. In the meantime, DPC data may be useful to evaluate prevention strategies against AHT in Japan.
